# Comparing oral health in patients with different levels of dental anxiety

**DOI:** 10.1186/s13005-018-0182-4

**Published:** 2018-11-20

**Authors:** Alexander Zinke, Christian Hannig, Hendrik Berth

**Affiliations:** 1 Technische Universität Dresden, Carl Gustav Carus Faculty of Medicine, Division of Psychological and Social Medicine and Developmental Neurosciences, Research Group Applied Medical Psychology and Medical Sociology, Fetscherstr 74, 01307 Dresden, Germany; 2 Universitätsklinikum Carl Gustav Carus Dresden, Policlinic of Dental Maintenance, Fetscherstr 74, 01307 Dresden, Germany

**Keywords:** Dental anxiety, Oral health, Medical Psychology

## Abstract

**Background:**

Dental Anxiety is still today one of the most common fears and is therefore a great challenge for every dental practitioner. The aim of this study was to identify patients with dental anxiety using the Dental Anxiety Scale and comparing different levels of dental anxiety with oral health using DMF-T and DMF-S index.

**Methods:**

This study questioned 1549 patients over the course of three years (2002–2005). DAS questionnaires were handed out before treatment and the state of oral health was evaluated using DMF-T and DMF-S.

**Results:**

There is no significant relation between high anxiety and the global DMF-T Score (*p* = 0.237), missing teeth (*p* = 0.034) and filled teeth (*p* = 0.237). There is however a significant increase in destroyed teeth, the higher the level of dental anxiety in the patient (*p* < 0.0001). There is as well a significant relationship between the global DMF-S Score (*p* = 0.042) and dental anxiety. No relationship was found comparing missing surfaces (*p* = 0.107) and filled surfaces (*p* = 0.516) with dental anxiety. Destroyed 16 surfaces are, however, significantly higher in patients with more dental anxiety (*p* < 0.0001). A higher dental anxiety therefore often causes minimalistic dentistry to fail due to more teeth being destroyed.

**Conclusions:**

Patients with dental anxiety still have a worse oral hygiene than patients without dental anxiety. It is still necessary, in this time of caries prevention rather than over-treatment, to be educated so that patients suffering dental fear receive the right treatment.

## Background

Dental anxiety is despite further medical advances a very common disorder in the general population. Nearly 80% of all adults in industrial countries feel discomfort before dental treatment, 20% state to be scared of dental treatment and 5% evade dental treatment fully [1]. It has been stated as the fifth most common fear by Agras [2]. The prevalence of dental anxiety can be seen across all age groups. Even young children are observed to have an avoidance behaviour towards dental treatment, which can be linked to parents influences [3]. A study conducted by Hakeberg singled out 20–39 year old patients to be at the highest risk of having or obtaining anxiety before dental treatment [4]. This agrees with the theory of aging reducing the presence of anxiety disorders and fears [5]. The difficulty of treatment derives in the factors of onset of dental anxiety being different for every age group. Child-hood dental anxiety is strongly influenced by exogenous sources such as one or more members of the family, adolescent derived anxiety is characterized by trait-anxiety and adults by the presence of multiple fears and symptoms indicative of psychiatric problems [6]. It is however vital not to confuse discomfort before treatment with a fully developed dental anxiety disorder. The fear of a certain situation is present in every individual and defines an interindividual stable, however interindividual varying trait to judge a certain known situation as threatening [7]. Not every sign of anxiety during dental treatment describes an anxiety disorder. Sartory et al. [8] conducted a study presenting sounds, hearable during a dental examination, to patients with diagnosed high dental anxiety and those without a diagnosis. Both groups judged dental sounds significantly more aversive than neutral sounds (bird twitter). A habitual fear of dental procedures begins before and not as a reaction after treatment [9]. An extreme condition of dental anxiety can be described as a dental phobia. Phobias can be classified using the International Classification of Diseases (ICD). A phobia is, according to the ICD-10 Chapter V, F40.0, a type of anxiety disorder. It is classified as an irrational fear of a defined, generally non-dangerous situation which is avoided fully or endured with great distress [10]. It is fully separated from phobias of certain stimuli during dental treatment, such as injections [11]. A possibility to differentiate between the stages of anxiety and phobia is the impact it has on the patients everyday routine and life. If it interferes with the persons social life, occupation and has an effect on normal functioning, it can be considered a specific (dental) phobia [12]. Weiner and Sheehan [13] were able to describe two different origins of dental anxiety, deriving from questionnaires specifically handed out about dental treatment. Exogenic dental anxiety is a conditioned phobia as a result of negative experiences during dental treatment. The endogenic dental anxiety is part of a generalised anxiety disorder with multiple phobias and psychiatric diagnoses. Dental anxiety is stressful for the patient as well as the dentist due to reduced cooperation, requirement of more time and an unpleasant environment [14]. This may even lead to misdiagnosis and therefore mistreatment such as the analysis of tooth vitality [15]. Patients avoiding treatment fully results in bad dental and periodontal health [16]. These patients may visit a dental clinic only when pain begins to feel unbearable requiring complicated procedures such as endodontic treatment or tooth removal. This vicious circle denies a healthy patient-dentist relationship [17]. In this study our aim was to identify patients with dental anxiety using the Dental Anxiety Scale [18] and assessing their oral health using the DMF-T and DMF-S index and comparing these results with patients having no dental anxiety.

## Method

In Dresden, Germany, the research group for Medical Psychology and Medical Sociology collected data over the course of 3 years (2002–2005). DAS questionnaires were handed out to 1549 patients before treatment. All patients needed to be 18 years old and had to voluntarily take part in this study. They were required to complete our questionnaires before treatment in a dental clinic. Other inclusion criteria were a sufficient knowledge of the German language, the physical and mental ability to complete the questionnaires, oriented as to time and place as well as no display of psychiatric symptoms. All patients gave written informed consent, and only patients providing written informed consent were included as study participants. During treatment the patients’ oral health was assessed by documenting the number of destroyed, missing and filled teeth/surfaces (DMFT/DMF-S) using visual aid and x-rays if present. DMF-T scores of *N* = 881 patients and DMF-S scores of *N* = 602 patients were allocatable. All results were analysed using the statistics software IBM SPSS V24. Mean total values were analysed using One-Way-ANOVA. Normality is not given for every parameter. One-Way ANOVA is, however, resistant to a violation of normality as proven in many studies, especially with a large sample as it is used here [19–22].

In this study, *P* values less or equal to 0.05 were statistically significant. This research was directed using STROBE guidelines.

### Dental anxiety scale

This self-assessing questionnaire is the most common instrument used in research of dental anxiety. It consists of four Items related to a dental situation [18]. The patient needs to judge himself as in how anxious he feels during these described circumstances on a scale of 1 (low anxiety) to 5 (highly anxious). Corah et al. [23] described a mean average score of 9.1 in a population of 2103 people. A cut-off value of 15 separates those with dental anxiety from those feeling slightly anxious scoring 13 to 15 points, as well those with no anxiety below a score of 13. The Retest-reliability was described as rtt = 0.86 [24]. Cronbach’s alpha as an estimate of reliability is 0.80 [37]. In this study Cronbachs Alpha were calculated .885 (*N* = 1550).

### DMF-t/DMF-s

The Decayed, Missing, Filled – index has been a standardized measurement of oral health for over 70 years [25]. The DMF-index is applied to the permanent dentition when written in capital letters, an as dmf-index as a variation for primary dentition. Applied to Teeth, the DMF-T index has a score range of 0–32 if including the third molars. A DMF-T index below 1.2 is seen as very low, 1.2–2.6 as low, 2.6–4.4 as mediocre and above 4.5 as high. The DMF-S index applies to tooth surfaces and the score ranges from 0 to 148, if including the third molars. For posterior teeth, five surfaces are examined: facial, lingual, mesial, distal and occlusal. On anterior teeth there are four surfaces: facial, lingual, mesial, distal. When a carious lesion and a filling is present on a surface, the surface is marked as D (destroyed). When the tooth has been extracted due to caries the surface is listed as M (missing) and if there is a filling present on the surface it is counted as F (filled). Teeth extracted for any other reasons than tooth decay, such as orthodontics, are not included as missing [26].

## Results

A total of 1549 patients returned their questionnaires. Out of these patients 865 (55.8%) were female and 684 (44.2%) male. Out of the 881 patients used in our DMF-T database, 415 (47.1%) were male and 466 (52.9%) female. The 602 patients completing our DMF-S scores included 299 (49.7%) males and 303 (50.3%) females. The youngest patients were 18 and the oldest patients 88 years old with a mean average age of 45.7 years (SD = 18.7).

### Association of DMF-T values with dental anxiety

The results of destroyed, missing and filled teeth, collected during the examination of patients was added together and mean average values were calculated for each anxiety group determined by DAS questionnaires (Table 1). It can be observed that there is a slight, however not statistically significant, increase in the DMF-T average score the more anxiety the patient states to have.

**Table 1:** Different anxiety groups compared to DMF-T values (*M*, *SD*, One-way-Anova)

	*N*	DMF-T global value *M* (*SD*)	Destroyed *M* (*SD*)	Missing *M* (*SD*)	Filled *M* (*SD*)
Low anxiety	590	14.20 (8.23)	1.34 (2.45)	4.73 (6.42)	8.17 (5.61)
Moderate anxiety	243	15.02 (7.86)	1.89 (3.01)	6.14 (9.83)	7.44 (5.36)
High anxiety	47	15.72 (7.91)	2.70 (3.24)	6.13 (6.42)	6.63 (5.41)
One-way ANOVA:		*F*(2, 877) = 1.441, *p* = 0.237, Eta^2^ = 0.003	*F*(2, 876) = 8.102, *p* < 0.0001, Eta^2^ = 0.018	*F*(2, 875) = 3.407, *p* = 0.034, Eta^2^ = 0.008	*F*(2, 875) = 2.769, *p* = 0.237, Eta^2^ = 0.006

In Table 1 we tried to find out if there are significant correlations between the individual categories of the DMFT index and dental anxiety. Patients with high anxiety had significantly more teeth destroyed by caries than those with low anxiety. Missing teeth seem to increase with moderate anxiety compared to low anxiety. The difference between patients with moderate anxiety and high anxiety is smaller. The increase of missing teeth due to tooth decay in patients the more anxiety they judge themselves with was found to be statistically significant. The number of filled teeth decreased the more anxiety the patient had. It could however not be proven as significant.

### Association of DMF-S values with dental anxiety

We furthermore compared the specific surfaces of each tooth for every patient and calculated an average value of its destroyed, missing or filled condition. It is visible in Table 2 that the mean average score of the DMF-S increases the higher the anxiety of the patient is, this means that patients with more anxiety have more surfaces either destroyed, missing or filled. Table 2 shows the individual categories of the DMF-S index compared to the anxiety groups determined through the Dental Anxiety Scale. Patients with low dental anxiety showed to have less destroyed surfaces in average than those with moderate and high anxiety There was a strong statistical correlation between these. The mean average values for missing teeth increase the more anxiety the patient has. Patients with low anxiety show a lower mean average of missing surfaces, whereas those with high anxiety show a higher mean value. This was however not proven to be statistically significant. The number of filled surfaces decreases the higher the anxiety of the patient. A patient with low anxiety has in average more filled surfaces while a patient with high anxiety.

**Table 2:** Different anxiety groups compared to DMF-S values (*M*, *SD*, One-way-Anova)

	*N*	DMF-S global value *M* (*SD*)	Destroyed *M* (*SD*)	Missing *M* (*SD*)	Filled *M* (*SD*)
Low anxiety	418	52.41 (35.33)	3.16 (5.62)	18.44 (25.93)	27.94 (22.03)
Moderate anxiety	157	57.48 (35.51)	4.88 (6.80)	21.73 (30.45)	25.83 (19.59)
High anxiety	26	67.96 (33.78)	7.68 (11.86)	28.80 (31.75)	25.36 (19.34)
One-way ANOVA:		*F*(2, 598) = 3.180, *p* = 0.042, Eta^2^ = 0.011	*F*(2, 596) = 9.189,*p* < 0.0001, Eta^2^ = 0.030	*F*(2, 596) = 2.245, *p* = 0.107, Eta^2^ = 0.007	*F*(2, 596) = 0.662,*p* = 0.516, Eta^2^ = 0.002

## Discussion

Dental anxiety is a complex psychological inhibitor which may even influence other parts of the individuals life. It is necessary to analyse how to best treat patients with high dental anxiety to prevent bad oral health. The large sample size of a general population used in this study makes this study especially relevant for dental practitioners. The DMF-T index was not significantly linked to dental anxiety, even though there is a slight increase the DMF-T average score visible. It was possible to link a larger amount of destroyed and missing teeth to patients with a higher amount of anxiety before a dental visit. These patients make a dental appointment not as regularly and therefore there is no feed-back from a professional to diagnose and treat carious lesions in their early stages to prevent tooth decay. Dentists have the obligation to educate their patients in how to improve oral health if necessary. This is not possible if no regular check-ups are appointed. Dental anxiety can cause a patient to evade dental treatment fully, even if pain is present. Every patient visiting our dental clinics had to overcome himself and make an appointment to be part of our study. This could still indicate that there could be a group of highly anxious patients which would feel too worried to make an appointment for a dental treatment, let alone take part in our study during a dental examination which might propose even more stress [27]. When it becomes unbearable the dental appointment might be too late and end up in tooth removal or invasive tooth preparation. The amount of filled teeth could not be associated with dental anxiety like in similar studies [26], even though there is a decline in filled teeth noticeable the more anxiety the patients admit having. The DMF-S index can be significantly connected to dental anxiety. Patients with a higher amount of dental anxiety have a higher average of destroyed, missing and filled surfaces than patients without or little dental anxiety. The same was observed for destroyed surfaces as well as missing surfaces. Both were significantly higher in patients with dental anxiety. Our thesis that patients with a higher anxiety of dental treatment have worse oral hygiene than patients which do not have this level of anxiety was therefore partially confirmed.Corah’s Dental Anxiety Scale is a validated and frequently used instrument to determine dental anxiety in a population [18]. There have however been concerns indicating that no question about anaesthesia is present. The sight and thought of needles is one of the most fear inducing stimuli during dental treatment [28]. This item is included in the Modified Dental Anxiety Scale created by Humphris, Morrison and Lindsay [29]. Using this item however generalises the fear of needles and the fear of dental treatment, according to ICD-10 two separate, even though connected phobias. In addition, it has been proven that values acquired by DAS and MDAS are very similar [30]. We achieved the expected results using the DMF-T and DMF-S index. While being powerful, they do hold certain boundaries. The data is acquired solely using a probe, mirror and cotton rolls, it has been found to exist inter-observer bias when detecting carious lesions [31]. It is also not possible to identify the patients’ treatment needs or teeth at risk. The dentist faces a difficulty when identifying how the missing teeth occurred. A tooth removed due to an accident or orthodontic treatment should not have an influence on an index for oral health. If no previous dental records exist, the dentist has to rely on the information given by the patient. This study was limited by not including the patients socio-economic status as well as the lack of documentation of oral hygiene habits. It would have been interesting to find out how often and how patients with high DMF-T values care for oral health each day, as well as the budget and education influencing it. The awareness of taking part in a study, even if anonymous, might encourage to give false information to make the outcome more favourable for the patients’ self. This study shows the necessity of better education for patients before dental fear develops and if already present. Patients with dental anxiety still have a worse oral hygiene than patients without dental anxiety. A high amount of destroyed and missing teeth limits the possibilities of dental treatment and denies a minimal-invasive dentistry which can be quick and painless. Screening of patients should be mandatory before treatment to prepare the professional and allow him to enlighten possible patients with anxiety. An early screening in kindergartens would be useful to educate children as well as parents. Patients with high amounts of dental fear should have a state-controlled telephone number with educated practitioners to inform themselves anonymously about any concerns before treatment.

## Conclusion

The large sample of dental patients in this study determined strong associations. There is a significant increase in the DMF-S index and dental anxiety. Additionally, patients with dental anxiety have more destroyed teeth and destroyed surfaces than patients without dental anxiety.
